# Supplementary material for: “I Need Someone to Help Me Build Up My Strength”: A Meta-Synthesis of Lived Experience Perspectives on the Role and Value of a Dietitian in Eating Disorder Treatment
Source: Behav Sci (Basel). 2023 Nov 17;13(11):944. doi: 10.3390/bs13110944 (PMC10669240; doi:10.3390/bs13110944)
Supplement: Supplementary file 1 [file behavsci-13-00944-s001.zip › Supplementary file S1 - Search strategy.pdf]

Supplementary file S1

"I Need Someone to Help Me Build Up My Strength": A Meta-Synthesis of Lived Experience Perspectives on the Role and Value of a Dietitian in Eating Disorder Treatment

Yive Yang [1], Janet Conti [1,2], Caitlin M. McMaster [3,4,5,6], Milan K. Piya [1,7] and Phillipa Hay [1,7,*]

[1] Translational Health Research Institute, School of Medicine, Western Sydney University, Campbelltown, NSW 2560, Australia; yive.yang@gmail.com (Y.Y.); j.conti@westernsydney.edu.au (J.C.); m.piya@westernsydney.edu.au (M.K.P.)
[2] School of Psychology, Western Sydney University, Penrith, NSW 2750, Australia
[3] Sydney School of Health Sciences, Faculty of Medicine and Health, University of Sydney, Camperdown, NSW 2050, Australia; caitlin.mcmaster@sydney.edu.au
[4] Eating Disorder and Nutrition Research Group (ENRG), Translational Health Research Institute, Faculty of Medicine, Western Sydney University, Campbelltown, NSW 2560, Australia
[5] South Western Sydney Local Health District, Liverpool, NSW 2170, Australia
[6] Ingham Institute for Applied Medical Research, Liverpool, NSW 2170, Australia
[7] Camden and Campbelltown Hospitals, South Western Sydney Local Health District, Liverpool, NSW 2560, Australia
* Correspondence: p.hay@westernsydney.edu.au

Table S1. Search strategy used on the Ovid MEDLINE(R) ALL platform.

1	exp "Feeding and Eating Disorders"/
2	(eating disorder* or disordered eating or anorexi* or bulimi* or binge or feeding disorder* or orthorexia or muscle dysmorphia or rumination disorder* or purging disorder* or night eating syndrome or intake disorder* or ARFID or EDNOS or OSFED or UFED or PICA).ti. or (eating disorder* or disordered eating or anorexi* or bulimi* or binge or feeding disorder* or orthorexia or muscle dysmorphia or rumination disorder* or purging disorder* or night eating syndrome or intake disorder* or ARFID or EDNOS or OSFED or UFED or PICA).ab.
3	1 or 2
4	Dietetics/ or Nutritionists/
5	(dieti#ian* or dietetic* or nutrition*).ti. or (dieti#ian* or dietetic* or nutrition*).ab.
6	4 or 5
7	nutrition therapy/ or diet therapy/ or nutritional support/ or professional role/ or "scope of practice"/
8	(treatment* or counsel* or therap* or intervention* or care or consult* or recommend* or plan or advice or management or educat* or prescri* or support or role* or function*).ti. or (treatment* or counsel* or therap* or intervention* or care or consult* or recommend* or plan or advice or management or educat* or prescri* or support or role* or function*).ab.
9	7 or 8
10	3 and 6 and 9
