# Supplementary material for: “I Need Someone to Help Me Build Up My Strength”: A Meta-Synthesis of Lived Experience Perspectives on the Role and Value of a Dietitian in Eating Disorder Treatment
Source: Behav Sci (Basel). 2023 Nov 17;13(11):944. doi: 10.3390/bs13110944 (PMC10669240; doi:10.3390/bs13110944)
Supplement: Supplementary file 1 [file behavsci-13-00944-s001.zip › Supplementary file S2 - Full-text inclusion and exclusion criteria.pdf]

Supplementary file S2

"I Need Someone to Help Me Build Up My Strength": A Meta-Synthesis of Lived Experience Perspectives on the Role and Value of a Dietitian in Eating Disorder Treatment

Yive Yang [1], Janet Conti [1,2], Caitlin M. McMaster [3,4,5,6], Milan K. Piya [1,7] and Phillipa Hay [1,7,*]

1 Translational Health Research Institute, School of Medicine, Western Sydney University, Campbelltown, NSW 2560, Australia; yive.yang@gmail.com (Y.Y.); j.conti@westernsydney.edu.au (J.C.); m.piya@westernsydney.edu.au (M.K.P.)
2 School of Psychology, Western Sydney University, Penrith, NSW 2750, Australia
3 Sydney School of Health Sciences, Faculty of Medicine and Health, University of Sydney, Camperdown, NSW 2050, Australia; caitlin.mcmaster@sydney.edu.au
4 Eating Disorder and Nutrition Research Group (ENRG), Translational Health Research Institute, Faculty of Medicine, Western Sydney University, Campbelltown, NSW 2560, Australia
5 South Western Sydney Local Health District, Liverpool, NSW 2170, Australia
6 Ingham Institute for Applied Medical Research, Liverpool, NSW 2170, Australia
7 Camden and Campbelltown Hospitals, South Western Sydney Local Health District, Liverpool, NSW 2560, Australia
* Correspondence: p.hay@westernsydney.edu.au

Table S2. Full-text inclusion and exclusion criteria.

Inclusion Criteria	Exclusion Criteria
1) qualitative or mixed methods research design containing qualitative findings;<br>2) focused partially or exclusively on the role of a dietitian in treatment for any ED (including but not restricted to anorexia nervosa (AN), atypical anorexia nervosa (AAN) bulimia nervosa (BN), binge eating disorder (BED), other specified feeding or eating disorder (OSFED), eating disorder not otherwise specified (EDNOS), avoidant/restrictive food intake disorder (ARFID), and orthorexia nervosa); and<br>(3) were from the perspective of individuals with lived experience of an ED.	1) were not about a dietitian or dietetic treatment as it pertains to ED care;<br>2) did not distinguish data regarding a dietitian from treatment provided as part of a multi-disciplinary team (MDT);<br>3) were from the perspective of healthcare professionals or dietetic students;<br>4) focused only on medical or pharmacological interventions;<br>5) were not original research;<br>6) were an abstract only;<br>7) were a study protocol;<br>8) were an earlier version of an included paper; or<br>9) were not written or translated into English.
