# Supplementary material for: “I Need Someone to Help Me Build Up My Strength”: A Meta-Synthesis of Lived Experience Perspectives on the Role and Value of a Dietitian in Eating Disorder Treatment
Source: Behav Sci (Basel). 2023 Nov 17;13(11):944. doi: 10.3390/bs13110944 (PMC10669240; doi:10.3390/bs13110944)
Supplement: Supplementary file 1 [file behavsci-13-00944-s001.zip › Supplementary file S3 - Quality appraisal.pdf]

Supplementary file S3

“I Need Someone to Help Me Build Up My Strength”: A Meta-Synthesis of Lived Experience Perspectives on the Role and Value of a Dietitian in Eating Disorder Treatment

Yive Yang [1], Janet Conti [1,2], Caitlin M. McMaster [3,4,5,6], Milan K. Piya [1,7] and Phillipa Hay [1,7,*]

[1] Translational Health Research Institute, School of Medicine, Western Sydney University, Campbelltown, NSW 2560, Australia; yive.yang@gmail.com (Y.Y.); j.conti@westernsydney.edu.au (J.C.); m.piya@westernsydney.edu.au (M.K.P.)
[2] School of Psychology, Western Sydney University, Penrith, NSW 2750, Australia
[3] Sydney School of Health Sciences, Faculty of Medicine and Health, University of Sydney, Camperdown, NSW 2050, Australia; caitlin.mcmaster@sydney.edu.au
[4] Eating Disorder and Nutrition Research Group (ENRG), Translational Health Research Institute, Faculty of Medicine, Western Sydney University, Campbelltown, NSW 2560, Australia
[5] South Western Sydney Local Health District, Liverpool, NSW 2170, Australia
[6] Ingham Institute for Applied Medical Research, Liverpool, NSW 2170, Australia
[7] Camden and Campbelltown Hospitals, South Western Sydney Local Health District, Liverpool, NSW 2560, Australia
* Correspondence: p.hay@westernsydney.edu.au

Table S3. Quality appraisal.

Author	1. Aims	2. Qualitative methodology	3. Research design	4. Recruitment	5. Data collection	6. Relationships	7. Ethical issues	8. Data analysis	9. Findings	10. Value
Bakland et al. (2019)	✓	✓	✓	✓	✓	✓	✓	✓	✓	✓
Blumenthal (2020)	✓	✓	✓	✓	✓	?	✓	✓	✓	✓
Bravender et al. (2017)	✓	✓	✓	✓	✓	×	✓	×	✓	✓
Darden et al. (2017)	✓	✓	✓	✓	✓	?	✓	✓	✓	✓
Elran-Barak et al. (2022)	✓	✓	✓	✓	✓	?	✓	✓	✓	✓
Heafala et al. (2022)	✓	✓	✓	✓	✓	✓	✓	✓	✓	✓
Lyons et al. (2018)	✓	✓	✓	✓	✓	✓	✓	✓	✓	✓
Marek (1995)	✓	✓	✓	✓	✓	✓	✓	✓	✓	✓
Munro et al. (2014)	✓	✓	?	✓	?	?	×	?	✓	✓
Petry (2017)	✓	✓	✓	✓	✓	?	✓	✓	✓	✓

Reyes-Rodriguez (2016)	✓	✓	✓	✓	✓	?	✓	?	✓	✓
Roots et al. (2009)	✓	✓	✓	✓	✓	?	?	✓	✓	✓
Taylor et al. (2021)	✓	✓	✓	✓	✓	?	?	✓	✓	✓
Thompson (2007)	✓	✓	✓	✓	✓	?	✓	✓	✓	✓
Woodruff et al. (2020)	✓	✓	✓	✓	✓	✓	✓	✓	✓	✓

NB: ✓ denotes this criterion was met; × denotes this criterion was not met; ? denotes that there was not enough evidence provided to assess this criterion. Full questions from the CASP: (1) Was there a clear statement of the aims of the research? (2) Is a qualitative methodology appropriate? (3) Was the research design appropriate to address the aims of the research? (4) Was the recruitment strategy appropriate to the aims of the research? (5) Was the data collected in a way that addressed the research issue? (6) Has the relationship between researcher and participants been adequately considered? (7) Have ethical issues been taken into consideration? (8) Was the data analysis sufficiently rigorous? (9) Is there a clear statement of findings? (10) How valuable is the research?
