# Supplementary material for: “I Need Someone to Help Me Build Up My Strength”: A Meta-Synthesis of Lived Experience Perspectives on the Role and Value of a Dietitian in Eating Disorder Treatment
Source: Behav Sci (Basel). 2023 Nov 17;13(11):944. doi: 10.3390/bs13110944 (PMC10669240; doi:10.3390/bs13110944)
Supplement: Supplementary file 1 [file behavsci-13-00944-s001.zip › Supplementary file S4 - Summary of included studies and their themes.pdf]

Supplementary file S4

"I Need Someone to Help Me Build Up My Strength": A Meta-Synthesis of Lived Experience Perspectives on the Role and Value of a Dietitian in Eating Disorder Treatment

Yive Yang [1], Janet Conti [1,2], Caitlin M. McMaster [3,4,5,6], Milan K. Piya [1,7] and Phillipa Hay [1,7,*]

[1] Translational Health Research Institute, School of Medicine, Western Sydney University, Campbelltown, NSW 2560, Australia; yive.yang@gmail.com (Y.Y.); j.conti@westernsydney.edu.au (J.C.); m.piya@westernsydney.edu.au (M.K.P.)
[2] School of Psychology, Western Sydney University, Penrith, NSW 2750, Australia
[3] Sydney School of Health Sciences, Faculty of Medicine and Health, University of Sydney, Camperdown, NSW 2050, Australia; caitlin.mcmaster@sydney.edu.au
[4] Eating Disorder and Nutrition Research Group (ENRG), Translational Health Research Institute, Faculty of Medicine, Western Sydney University, Campbelltown, NSW 2560, Australia
[5] South Western Sydney Local Health District, Liverpool, NSW 2170, Australia
[6] Ingham Institute for Applied Medical Research, Liverpool, NSW 2170, Australia
[7] Camden and Campbelltown Hospitals, South Western Sydney Local Health District, Liverpool, NSW 2560, Australia
* Correspondence: p.hay@westernsydney.edu.au

Table S4. Summary of included studies and their themes.

Paper	Theme	Subtheme
Bakland, 2019	Perceptions of impact	Obtaining tools
		Developing new perspectives
	Perceptions of treatment organisation and completion	Planning and framing
		Therapists' competence
Blumental, 2020	Weight-biased nutrition treatment experiences	Weight-cantered nutrition treatment
		Nutrition provider views about higher weight
		Thin provider phenotype
	Weight-inclusive nutrition treatment experiences	Health At Every Size® and Intuitive Eating nutrition approaches
		Nutrition provider validation of patient's eating disorder
		Nutrition provider acknowledgement of thin privilege
	Participant ideas for improved future support	Provider should acknowledge own privilege
		More diverse eating disorder providers
Bravender, 2017	Appreciation for having a respite from meal planning/responsibility	
Darden, 2017	Facilitators to forming a therapeutic working alliance with an RDN	Personality characteristics
		Emotional support
		Collaborative decision making
		Client-centered approach
		Self-disclosure
		Expertise/knowledge
	Barriers to forming a therapeutic working alliance with an RDN	Disregard
		Personality characteristics
		Nature of the eating disorder
		Nature of the dietitian's role
		Assumptions
		Lack of proper support

		Poor communication
		Power struggle
Elran-Barak (2021)	Silent dialogue: Talking is useless (client's position) - This position describes users' unwillingness to be truly engaged in a dialogue with their rds. Users described that they wanted to remain in full control of their ED; therefore, they did not want their RD's involvement.	
	Acting out: Cancelation and deception (client's position) - describes the occasions in which users felt that it was not possible to resolve their disagreements with their RDs through genuine dialogue. In these occasions, women saw their relationship with the RD as a war they needed to win rather than as a cooperative relationship	
	Tough love: Firm boundaries accompanied by empathy (RD's position) - RDs frequently responded by setting firm and clear boundaries	
	Containment: Concern and understanding (RD's position) - includes descriptions of caring and supportive RDs who created a containing atmosphere: that is, RDs who took it upon themselves to go the extra mile and make clients feel seen and understood	
Heafala, 2022	Valuing a person-centered approach to dietetic care	Connecting with the individual and adapting care to the needs of consumers
		Empowering consumers and carers with knowledge and skills to navigate recovery
		Taking a holistic and inclusive approach to dietetic care
		Perceiving communication and coordination between treating team, consumers and carers as beneficial for recovery
		Dietitians' understanding, experience and expertise in eds
	The therapeutic alliance is central to engaging in dietetic care	Consumers perceiving mutual trust, empathy and rapport as integral to therapeutic alliance
		Accountability, support and validation perceived as important for ongoing dietetic care
	Sharing the complex journey	Carers recognizing the signs and symptoms and advocating for help
		Consumers and dietitians needing patience and perseverance, and trusting the process of recovery
		Choosing recovery and having a support system
		Dietitians considering impact on families and reinforcing role of nutrition in recovery
		Prioritizing and balancing treatment with daily life
Lyons, 2018	The dietician/nutritionist was almost expecting to work with someone who both physically	

	and mentally wanted to eat and therefore provided general information on what to eat and when, but not on how to actually eat or to work with the men’s individual issues.	
Marek, 1995	Biological components that contribute to the etiology, treatment, and outcomes for those experiencing eating problems – represented by nutritional counseling provided by a registered dietitian	Desired changes in eating patterns
		Need for general nutritional information
		Desire for personalized nutritional information
		Participant concerns
	"Having the three components together was most important?” - emotional, the physical, the social elements all addressed	
Munro, 2014	Staff perceived as supportive, caring and genuine	
	Patients valued a holistic psychological approach based on emotional and physical needs and not just weight	
	Patients valued individualised care	
Petry, 2017	Feelings and perceptions about eating behaviors after the anorexia nervosa experience	
Reyes-Rodriguez, 2016	Describing eating patterns	
	Emotional distress	
	Latino cultural values	
	Family conflicts associated with disturbed eating behaviors	
	Lack of knowledge of healthy eating	
	Treatment progress	
Taylor, 2021	Disconnects (between clinician and patient perspectives)	Isolated clinicians within treatment teams
		Separating physical and psychological dimensions
		Divergent orientations toward tracking
Roots, 2009	Importance and availability of dietetic therapy	
Thompson, 2007	Interpersonal relationships are perceived as Important in the recovery process	
Woodruff, 2020	Relying on the lifeline of communication	
	Supporting autonomy	
	Drawing on individual strengths	
	Valuing synergy	
