# Supplementary material for: “I Need Someone to Help Me Build Up My Strength”: A Meta-Synthesis of Lived Experience Perspectives on the Role and Value of a Dietitian in Eating Disorder Treatment
Source: Behav Sci (Basel). 2023 Nov 17;13(11):944. doi: 10.3390/bs13110944 (PMC10669240; doi:10.3390/bs13110944)
Supplement: Supplementary file 1 [file behavsci-13-00944-s001.zip › Supplementary file S5 - Additional exemplar extracts.pdf]

Supplementary file S5

"I Need Someone to Help Me Build Up My Strength": A Meta-Synthesis of Lived Experience Perspectives on the Role and Value of a Dietitian in Eating Disorder Treatment

Yive Yang [1], Janet Conti [1,2], Caitlin M. McMaster [3,4,5,6], Milan K. Piya [1,7] and Phillipa Hay [1,7,*]

[1] Translational Health Research Institute, School of Medicine, Western Sydney University, Campbelltown, NSW 2560, Australia; yive.yang@gmail.com (Y.Y.); j.conti@westernsydney.edu.au (J.C.); m.piya@westernsydney.edu.au (M.K.P.)
[2] School of Psychology, Western Sydney University, Penrith, NSW 2750, Australia
[3] Sydney School of Health Sciences, Faculty of Medicine and Health, University of Sydney, Camperdown, NSW 2050, Australia; caitlin.mcmaster@sydney.edu.au
[4] Eating Disorder and Nutrition Research Group (ENRG), Translational Health Research Institute, Faculty of Medicine, Western Sydney University, Campbelltown, NSW 2560, Australia
[5] South Western Sydney Local Health District, Liverpool, NSW 2170, Australia
[6] Ingham Institute for Applied Medical Research, Liverpool, NSW 2170, Australia
[7] Camden and Campbelltown Hospitals, South Western Sydney Local Health District, Liverpool, NSW 2560, Australia
* Correspondence: p.hay@westernsydney.edu.au

Table S5. Additional exemplar extracts.

Theme 1: *"guidance and structure"* – Provision of nutrition knowledge and skills		
**Sub-theme**	**Source**	**Extract**
Subtheme 1(a): Nutrition education	Bakland (2019)	• *"It is important to get the facts on the table about what can potentially happen to you if you keep hurting your body."* (Mary)<br>• *"I had difficulties realizing that I needed food. I did learn that one needs a rather large amount of food when one exercises the way I do. It actually made me change the way I view my diet."* (Julie)
	Darden (2017)	• *"I needed somebody to really tell me what a normal relationship with food really looked like and she did that"* (p.67)
	Marek (1995)	• *"Nutrition has helped me in that now I'm still learning how to balance what I eat. Eat more protein, and stuff like that. Like before, if you asked me what a vegetable was I knew kind of, but I would call fruits vegetables. I didn't know. I learned now what I kind of need to eat at each meal. When I'm hungry I've learned what I could pick, which would make me less hungry and feel better."* (p.79)

		• *"I labelled foods wrong, no you can't have that. Lately I've learned that I can't do that cause if you say something is a bad food then I am going to punish myself for eating it."* (p.79)<br>• *When I'm hungry I've learned what I could pick, which would make me less hungry and feel better.* (p.79)<br>• *"The nutritional therapy I really, really benefitted from because I never paid any attention to nutrition at all. And all of the sudden I started cutting out my fat, well that's wrong too cause (nutritionist) told me that I don't get much protein at all, so I can lose all the weight I want but if I don't get any protein, I'm losing muscle."* (p.79)<br>• *"I think she did a really good job, ... I went to a nutritionist before and, I guess, a typical nutritionist where she says eat this amount of this and this amount of that and this amount of that. Well, when I told (nutritionist) she was like, that's not the way that you have a healthy lifestyle, that's not something that you can live with ... I think that's a really easy way to adjust your eating habits healthfully but also live with that and change your life. You know change it, you know, not just for your diet."* (p.80)<br>• *"She just sort of reinforced to me that I did know what I should be doing. I just needed to get on the right track and do it. It was like the steps to recovery, she went over that and those helped a lot. Just knowing that it goes back and forth, it's not all just one straight line."* (p.80)<br>• *"For me the biggest thing for me is she helped me realize I do have a problem. Like everybody else is doing this thing. She gave me some nutritional information that I didn't want to hear."* (p.81-82)
Subtheme 1(b): Meal planning and monitoring	Bakland (2019)	• *"Four regular meals is my mantra now. I did not eat regularly before I started the treatment. It might not make much sense to others when I say: four regular meals, but when I say it I remember how I started crying thinking about adding an evening meal."* (Irene)<br>• *"I had lost the feeling of hunger and the feeling of being full and I needed guidance and structure around mealtimes. We carefully added one meal at a time and reflected on what we ate and how much. When I had a small setback, I needed to make use of what I had learned about putting together a meal. My focus on food is more absent now and I really just eat to function."* (Mary)
**Theme 2: *"having all my bases covered"* – Dietitians as part of a multidisciplinary team**		
**Sub-theme**	**Source**	**Extract**
Subtheme 2(a): Enhancing care through a combined front	Taylor (2021)	• *"[The nutritionist] will take my weight sometimes to make sure everything is staying the same and if there's stuff that she has concerns about, she will ask me to talk to my doctor [PCP] about it"* (Patient 10)<br>• *"It was obviously different when I was in [in-patient] treatment itself. There were team meetings where they [clinicians] would talk about us. But that's different [now] because they [clinicians] are all in different places and not connected. They only talk if they really, really need to"* (Patient 10)<br>• *"I know they email back and forth in order for me to get additional sessions, the doctor had to approve them for my thera-pist. So my therapist had to kind of send an update of how I was doing and she recommended, you know, continuing"* (Patient 2)
	Woodruff (2020)	• *"I feel like they have literally saved my life. I think I think there's so much more you can capture by coordinating care because you're not just seeing one provider, you're getting that input from a whole team of people. The fact that they can*

		meet together and discuss your care, it just, makes such a difference. I feel like it's absolutely one of the most important things you can do in eating disorder recovery." (Kala)
Subtheme 2(b): Challenges with multi-disciplinary treatment	Marek (1995)	• *"I would want to meet with her more often. I know that she is very busy."* (p.83)<br>• *"The nutrition part, I think had to do with the fact that I didn't get as many meetings. She is very busy and I understand that."* (p.149)
	Woodruff (2020)	• *"And so, I think that the most valuable thing was that all of my providers rallied around that goal for me, and there were definitely rules in place . . . I think the learning experience from that and why I ended up, I stopped treatment for a while and then I went back to it, was just that I realized that providers would align with my goals, and what I wanted for my-self, and would, I felt like people would take that into account when they would work with me."* (Danielle)
**Theme 3: Challenges in nutritional treatment**		
**Sub-theme**	**Source**	**Extract**
Subtheme 3(a): Challenges with work-ing with ambivalence	Darden (2017)	• *"the eating disorder was just really loud at that time and I don't think it was anything that [my dietitian] did...if anyone had asked me if I had used behaviors while I was gone I would have said no."* (p.54)<br>• *"she was trying to set unrealistic goals with me"* (p.68)<br>• *"it made me feel like the dietitian and my mom were on a team against me"* (p.68)<br>• *"the dietitian came to the appointment with a defensive stance, as if they had an agenda, when you sense that immedi-ately barriers come up" "I had to turn over the control that I had to them"* (p.68)<br>• *"the eating disorder was just strong enough to prevent me from being honest"* (p.68)
	Elran-Barak (2021)	• *"My dietitian tells me that my weight is not going to go up forever, and that I gained weight because my weight needs to get balanced around my target weight. But I don't want this target weight and I don't want it to balance. I don't want to eat and I don't understand why I need to gain weight in order to recover."* (p.228)<br>• *"So tomorrow I meet with the dietitian and of course I know what she is going to say, and she will try to remind me of the price of starvation, and of how many times I've tried to starve myself in the past but couldn't keep it up, and she'll tell me that I must know I'm inviting the next binge, which will surely arrive. And I'm going to tell her that of course I know all of this, but what does she want me to do about it? I am not able to eat normally. I cannot. Just not. I either gulp it all up or starve. The middle line is foreign to me. I've tried but it doesn't work."* (p.228)<br>• *"I schedule an appointment for the dietitian and then cancel it the day before, time and time again. I cannot allow some-one else to see how much I weigh, to under- stand how fat I am, to make me see that it is real." (p.229)*<br>• *"I tricked my dietitian today. I ate before she weighed me and I used some other techniques that I cannot share [...] so I was at the lower boundary of my OK weight. I am so happy!"* (p.229)<br>• *"The dietitian told me to do a food log. I will try to do it but I don't really want her to know exactly what I am eating."* (p.229)
	Lyons (2018)	• *"There were times that she would talk to you, if you could imagine going weeeeee here comes the aeroplane as the spoon comes towards your mouth and I thought 'you have completely misunderstood me completely' and we just did not get on. I called her a tree hugging hippy. I come from a slightly scientific background that I like to know why, and I ended up having quite a heated argument."* (Russell)

	Petry (2017)	• *"For me it was absurd. But I decided to try only to follow 50% of it"* (Laura)
Subtheme 3(b): Varied dietitian knowledge and skills in working with EDs	Blumenthal (2020)	• *"My one nutritionist is the only one I really discussed [my restrictive eating behaviors and use of diet pills] with and she just said, 'It was very unhealthy.'"* (Participant 6)<br>• *"They did all these body tests, with calipers, and stuff, and this weight range. 10-pound weight range...I needed to be between 161 and 171 pounds. Which –<br>Interviewer: And, you entered treatment at around 160?<br>Abby: Yeah. Well, I was around 161, I think, so I was around the weight range. Interviewer: So, they didn't account for any refeeding weight gain?<br>Abby: No"* (Participant 8)<br>• *"...my treatment team's position was... 'You don't need to gain any weight. We just need to get you back to eating'..."* (Participant 19)<br>• *"... My friend[']s... plan of exchanges was dropped down really low because of weight gain, and I was like... 'why, why are you doing that?'"* (Participant 19)<br>• *"When I look at a provider's website and it's all really thin women... it's hard for me to trust that the [weight] bias isn't there.... [or] that [the] organization believes in a HAES® model... [if] they're not employing people at every size..."* (Participant 13)<br>• *"...Treatment providers [who] incorporated IE and HAES® [are] what led me to...full recovery."* (Participant 15)<br>• *"...When [my nutrition provider] said I was 'dieting like an anorexic,' [she] validated that the... pain that I was going through... the suffering I [endured] to achieve [weight loss] was definitely not normal."* (Participant 6)<br>• *"...When I was my sickest...[my dietitian] was urging me to go to treatment...which was really validating... everyone else in my life...was telling me how amazing [I looked]."* (Participant 8)<br>• *"...All ED treatment [centers should practice] HAES®... because it's harmful, [to] prescribe [on] one end of the weight spectrum what you're trying to cure [on] the other end of the weight spectrum..."* (Participant 9)
**Theme 4: *"it was my treatment and my recovery"* – Person-centred dietetic care**		
**Sub-theme**	**Source**	**Extract**
Subtheme 4(a): Building trust and connection	Darden (2017)	• *"a lot of what made it difficult was just that she was a dietitian...and I know that part of her job is to judge whether or not certain things were okay."* (p.55)<br>• *"recovery is not a linear path, so every time I come in and I've had a setback that week or haven't been doing as well, instead of trying to take me from where I was the week prior she's just like ok we can go from where you are now...she was very accepting in that way"* (p.67)<br>• *"she just took the time to get to know me well"* (p.67)<br>• *"He shared a little bit about himself"* (p.67)<br>• *"she disclosed to me that she was recovered from an eating disorder...I've definitely felt closer since then"* (p.67)<br>• *"that openness for [my dietitian] to say hey I'm not really sure what to do about this..."* (p.67)<br>• *"something about her vibe...I kind of perceived it as cold"* (p.68)<br>• *"she wasn't the right personality for me, she was too strong and mean for a kid like me"* (p.68)

		• *"it made me frustrated that even when I was trying so hard to be honest about everything that I wasn't being believed"* (p.68)<br>• *"she accused me of lying when I wasn't gaining weight, even though I was eating what she told me to...lying is very typical for people with eating disorders but I never lied to her"* (p.68)<br>• *"she said 'well the weight graph shows that [purging] is what you've been doing' but I wasn't"* (p.68)<br>• *"the fact that she was a dietitian and the one who would raise my intake levels"* (p.68)
	Elran Barack (2021)	• *"The dietitian who worked with me for more than a year left the clinic. In the last session, she did not even say goodbye. She was just busy typing on the computer and counting calories. I told her that I didn't want our last session to be just business as usual; I wanted to do something different for our last session. But she did not agree. She only said that I had to tell her if I wanted to continue with a substitute dietitian. I was really hurt. I feel as if I told her my most personal stories, and I gave away a part of my heart, and someone took it and threw it away into the trash."* (p.231-232)
	Heafala (2020)	• *"She was really empathetic and validating of my existing experiences and was very clear about I'm the expert of my own body and experience and that she was kind of co-working with me, rather [than] directing my input so it was very collaborative."* (P20)
	Lyons (2018)	• *"she feels more like a doctor so there isn't quite the relationship there"* (Russell)
	Marek (1995)	• *"I feel she knows me personally, not just talking to someone they don't know."* (p.82)<br>• *"I think it helped that she... takes notes on everything so they can come back and ask you questions about your life and it just seemed more personal. I think that helps - that helps me a lot."* (p.82)<br>• *She was more than a nutritionist, she was Mary Pat."* (p.82)
	Munro (2014)	• *"This is the first period [of treatment] I feel truly understood, and able to trust. . . SO much effort is made with me from everyone. I feel genuinely supported."* (p.222)
Subtheme 4(b): Collaborative care and goal setting	Darden (2017)	• *"She would address my concerns specifically and would work around solutions for them specifically"* (p.67)<br>• *"she wanted to put me back in control of my life in the right way"* (p.67)<br>• *"she would remind me that it was my meal plan, that it was my treatment and my recovery"* (p.67)<br>• *"it was the dietitian and I working as a team"* (p.67)<br>• *"she let me spread [snacks] out more so I wasn't feeling that all at once I was eating more than I wanted to eat"* (p.67)<br>• *"[my meal plan] was definitely more dictated to me"* (p.68)
	Heafala (2022)	• *"Looking back, I hated the dietitian appointments, at first, I hated them... I think I had to start to trust food... I kept saying to everyone, 'I just want to take baby steps'..."* (P16)
	Lyons (2018)	• *"I will explain to you why this is better for you, rather than I'm here to make you eat whatever you want to eat and don't panic about it"* (Russell)
	Munro (2014)	• *"Treatment is individualised and personal."* (p.223)
Subtheme 4(c): Dietetic treatment beyond nutrition	Bakland (2019)	• *"It was important for me that there was an expectation. First, there has to be an expectation from someone else, for me to fulfill, and then I have to learn to have expectations of my own."* (Monika)
	Darden (2017)	• *"we have contact between appointments, she told me I could text her...it's nice to have support when I get into stressful situations"* (p.67)<br>• *"it was always more or less, just in and out of those appointments, get my meal plan and go"* (p.68)

	Elran Barak (2021)	• *"I started weekly nutrition sessions. [...] Traveling an hour and a half on the roads [...] Traveling to be weighed, to cry, and to fall apart in someone's presence, – and it is not even her job to hold me. And then going back to school as if nothing happened. But I kept going. Something in that place signified a point of sanity to hold on to. One that [...] ultimately kept me alive. Kept me from going crazy. This was the first time that I did not drop out of treatment. I was proud of myself."* (p.231)<br>• *"Yesterday I was at the dietitian's and I was afraid. I had lost one kilo. I saw her disappointed look and I wanted to cry. I'm disappointed in myself as well. Now she wants me to come in once a week at the same hour each week, and after 5 sessions she's going to report to my medical doctor. I'm afraid I won't gain weight [...] I hope I gain some weight this week because I have a bad feeling that if I don't, they'll admit me to the hospital."* (p.230)
	Heafala (2022)	• *"She had such a firm, clear, understanding of the nuances of eating disorders and the complexities of eating disorders. I think for me that helped me build that sense of trust with her because she could really understand."* (P01)
	Lyons (2018)	• *"I can't email her in between and say I've just been told my bone density is through the floor I need to eat more calcium can you help me? You have to wait until the next session... It's a little bit more structured and a little bit too serious. It's more what I need!"* (Russell)
	Marek (1995)	• *"She just helped me realize that everybody has a different body type and not everyone is going to be this perfect size 5 or 6 that's a model or whatever and that to listen to your body when it's hungry and when its full."* (p.82)
